# Measurement-based dosimetric comparison of kilovoltage and megavoltage X-ray systems for static-field 3D conformal radiotherapy in companion animals

**DOI:** 10.4142/jvs.26032

**Published:** 2026-07-10

**Authors:** Yunji Lim, Geon Oh, Sun-Young Kim, Dongho Shin, Jaehwan Lee, Tae Hyun Kim, Sung Ho Moon, YoungMin Yun, Dong-Seok Shin

**Affiliations:** 1 College of Veterinary Medicine, Veterinary Medical Research Institute, Jeju National University, Jeju 63243, Korea.; 2 VIP Animal Medical Center, Seoul 06068, Korea.; 3 Proton Therapy Center, National Cancer Center, Goyang 10408, Korea.

**Keywords:** Radiation oncology, thermoluminescent dosimetry, anthropomorphic phantoms, treatment planning

## Abstract

**Importance:**

Kilovoltage (kV) X-ray radiotherapy systems have been introduced as a potential treatment option for companion animal radiotherapy, particularly for superficial targets. On the other hand, the lack of experimental validation of dose delivery accuracy has limited their broader clinical adoption.

**Objective:**

A measurement-based dosimetric comparison was made between kV and megavoltage (MV) X-ray systems.

**Methods:**

A 300 kV X-ray radiotherapy system and a 6 MV linear accelerator were evaluated for percent depth dose, penetration depth, beam profiles, and field size output factors. Eight static-field plans were generated for targets at varying depths and tissue densities in an anthropomorphic head phantom. Point doses were measured with thermoluminescent dosimeters and compared with treatment planning system-calculated doses. The calculated-to-measured absolute percentage differences were compared between the kV and MV systems using the Wilcoxon signed-rank test with a bootstrap 95% confidence interval.

**Results:**

Compared with the MV beam, the kV X-ray beam exhibited a negligible buildup distance, higher field-size dependence, a pronounced heel effect, and substantially lower penetration depth. Despite these dissimilarities, the absolute differences between the calculated and measured point doses were 0.1%–4.0% and 3.1%–6.5% for the kV and MV systems, respectively, across different target depths and tissue densities. The differences were significantly smaller for kV (median paired difference, −1.9 percentage points; 95% CI, −3.8 to −0.7; *p* = 0.0078).

**Conclusions and Relevance:**

Under the evaluated static-field phantom conditions, the commissioned kV treatment-planning model showed calculated-to-measured differences of 0.1%–4.0%. Although these values were lower than those for MV, this finding does not establish higher dosimetric accuracy for the kV system because measurement-related uncertainties may have contributed.

## INTRODUCTION

Radiation therapy is one of the main modalities for local tumor control, which aims to deliver the maximum doses to tumors while minimizing the doses to normal tissues. Veterinary radiation therapy has attracted increasing attention [1]. Currently, most veterinary hospitals that provide radiation therapy use megavoltage (MV) linear accelerators (LINACs), originally developed for human radiation therapy [2,3].

Modern MV LINACs equipped with advanced beam delivery techniques such as intensity-modulated radiation therapy (IMRT) and volumetric modulated arc therapy (VMAT) have enabled highly conformal dose delivery to deep-seated tumors [4,5,6]. Nevertheless, the buildup effect of MV X-ray beams, in which the dose maximum occurs below the skin surface, may reduce their dosimetric advantages for superficial tumors [7,8]. Therefore, kilovoltage (kV) X-ray beams, which can deliver a high surface dose, have been considered a potential option for treating superficial tumors in veterinary radiotherapy. Martin et al. [9] reported the clinical use of superficial kV radiotherapy in three dogs and one cat with cutaneous mast cell tumors of the head.

Although kV X-ray beams have been considered for superficial tumors [10], recent studies have also assessed their feasibility for deep-seated targets using human and animal models [11,12,13]. Bazalova-Carter et al. [11] reported that conformal dose distributions to deep-seated targets can be achieved using kV X-rays through an optimized arc therapy concept. They used Monte Carlo (MC) simulations to show that the dose conformity was comparable to that of a 15 MV VMAT plan. Seo et al. [12] performed dosimetric comparisons between kV X-ray (300 and 450 kV) and 6 MV X-ray treatment plans based on three-dimensional conformal radiotherapy (3DCRT) using a model-based dose calculation algorithm. Although optimal dose homogeneity was achieved with the 300 kV X-ray plan for a brain tumor case in a companion animal, the heel effect of the kV X-ray beams was not reflected in their beam data. Iseri et al. [13] confirmed the improved dosimetric accuracy of kV X-ray therapy for companion animals using MC-based dose calculations. Nevertheless, these studies have focused mainly on dose calculations or simulations, without measured dose data. In particular, MC simulations are typically performed under ideal conditions that may differ from those of real beam delivery conditions [14]. Therefore, experimental validation based on measured doses is required.

Recently, the LEP-300, a 300 kV X-ray therapy system for companion animals with a LINAC-like external design, has been commercialized as an advanced version of the previously proposed prototype [15]. This system enables 3DCRT planning and beam delivery. Hence, a comparison between calculated and measured doses can be performed using this system.

A measurement-based dosimetric comparison of kV and MV X-ray systems for static-field 3DCRT in companion animal radiotherapy was performed to address the limitations of previous studies. The comparison focused on the dosimetric characteristics at different depths and densities. This paper presents the experimental setup, measurement methods, and dosimetric comparison results.

## METHODS

This section is organized into three major sub-sections: 1) specifications of the radiotherapy systems used, 2) comparison of the basic beam characteristics, and 3) assessment of the dose delivery accuracy. The first sub-section describes the specifications of the kV and MV radiotherapy systems used in this study. The second sub-section focuses on comparing the basic beam characteristics of the two radiotherapy systems, such as percent depth dose (PDD), depth of penetration, field size output factor, and beam profiles. The last subsection evaluates the dose-delivery accuracy of each system by comparing the planned and measured point doses.

### Radiotherapy systems

#### LEP 300 system

The LEP 300 system (Auracare, Korea) used in this study is a companion animal radiation therapy system that utilizes kV X-rays generated at a tube voltage of 300 kVp (half-value layer: 3.15 mm Cu). The system has a LINAC-like design and includes hardware, such as a gantry, treatment couch, kV imager, and manually operated multi-leaf collimator (MLC), enabling 3DCRT (Supplementary Fig. 1A). AuraPlan ver. 1.0.3.3 (Auracare) was used as the treatment planning system (TPS). The LEP 300 system is self-shielded, eliminating the need for a LINAC vault, unlike human LINACs.

#### TrueBeam LINAC

The TrueBeam LINAC system (Varian Medical Systems, USA) was used for the dosimetric comparison with the kV X-ray system. This system can generate X-rays with energies of 6, 10, and 15 MV and also supports flattening filter-free X-ray beams, in which the flattening filter used to make the beam profile uniform is removed. The system is equipped with a gantry, treatment couch, automatic MLC, portal imager, and kV imaging system (such as X-ray imager and cone-beam computed tomography [CT]) (Supplementary Fig. 1B). Eclipse software 18.0.0 (Varian Medical Systems) was used as its TPS, which supports inverse planning algorithms. These features enable state-of-the-art techniques such as IMRT, VMAT, and respiratory-gated radiotherapy. Although 4 MV X-ray beams are commonly used in veterinary radiotherapy, the minimum photon energy available on the LINAC used in this study was 6 MV. Therefore, a 6 MV X-ray beam was used for the dosimetric comparisons.

### Comparison of basic beam characteristics

#### PDD and depth of penetration

The PDD, which represents the absorbed dose as a function of depth along the central axis of the beam, was measured for the kV and MV radiotherapy systems at field sizes of 4 × 4, 6 × 6, 8 × 8, 10 × 10, 12 × 12, and 15 × 15 cm^2^. The depths of dose penetration corresponding to 50%, 70%, and 90% of the maximum dose were derived from the measured PDDs. For these measurements, the detectors were mounted on a water phantom system (Blue Phantom; IBA Dosimetry, Germany) and aligned with the beam isocenter. A plane-parallel ionization chamber (Advanced Markus Chamber; PTW, Germany) was used for the kV X-ray beam due to negligible buildup distance (from the surface to the depth of maximum dose), whereas a compact cylindrical ionization chamber (CC13; IBA Dosimetry) was used for the MV X-ray beam. The kV and MV X-ray beams were delivered with source-to-surface distances (SSDs) of 60 and 100 cm, a tube current of 14 mA, and a dose rate of 500 monitor unit/min, respectively.

#### Beam profile

The beam profiles (lateral dose distribution measured at a specified depth) were measured using the same setup and beam conditions as the PDD measurements for the kV and MV X-ray beams. The measurements were taken by scanning the chamber in the inline and crossline directions while maintaining it at the depths of 10 cm for the MV X-ray beam and 2 cm for the kV X-ray beam, using a field size of 10 × 10 cm^2^. In these measurements, a compact cylindrical ionization chamber was used for the kV and MV X-ray beams. Compared with plane-parallel ionization chambers, this chamber had a smaller sensitive dimension in the lateral direction, which reduced the volume averaging effects and enabled accurate beam profile measurements without overestimating the penumbra (defined as the distance between the 80% and 20% dose levels across the dose gradient) [16].

The measured profiles were analyzed using the equations below to calculate the flatness and symmetry. The flatness was evaluated according to the AAPM TG-45 definition [17]. Symmetry was calculated using a TG-45-based formulation of the maximum deviation between symmetrically located points [17]. These calculations were performed within the region of interest (ROI), defined as the central 80% of the full width at half maximum (FWHM). In addition, the penumbra was calculated as follows:


$$\mathrm{Flatness}=\frac{D_{\max}-D_{\min}}{D_{\max}+D_{\min}}\times 100,$$

where *D_max_* and *D_min_* are the maximum and minimum doses within the central 80% of the FWHM of the dose profile, respectively.


$$\mathrm{Symmetry}={\mathrm{Max}}_{x\in ROI}\left|\frac{D\left(+x\right)-D(-x)}{D\left(+x\right)+D(-x)}\right|\times 100,$$

where *D*(+*x*) and *D*(−*x*) represent the doses at positions located symmetrically at a distance *x*, and the ROI corresponds to the central 80% of the FWHM of the dose profile.

#### Field size output factor

The field size output factors, defined as the ratio of the absorbed dose measured at the beam center for a specific field size to that measured for the reference field size, were measured for the kV and MV X-ray beams. The output factors were obtained for five field sizes: 3 × 3, 5 × 5, 7 × 7, 10 × 10, and 15 × 15 cm^2^. The measurements were taken using a Farmer-type ionization chamber (model 30013; PTW) under the same setup and beam conditions as those used for the PDD measurements, except for the chamber setup depth. The chamber was positioned at reference depths of 2 and 10 cm for kV and MV X-ray beams, respectively, as defined in the TG-61 [18] and TG-51 [19]/TRS-398 [20] reports. The same monitor units were used for all field sizes. The output factors were normalized to those obtained for a field size of 10 × 10 cm^2^.

### Assessment of dose delivery accuracy

The dose delivery accuracy was evaluated by measuring the point doses of the kV and MV X-ray beams using thermoluminescent dosimeters (TLDs) inserted into an anthropomorphic head phantom. This evaluation focused on the dependence on the target depth and surrounding tissue density. The measured doses were compared with the corresponding doses calculated using the TPS. The resulting absolute percentage differences were analyzed statistically to compare the two systems. The evaluation included CT imaging of the phantom, generation of the treatment plan, TLD calibration, and beam delivery under planned setup conditions. Further details are provided in the following sub-sections.

#### CT

Helical CT images of the anthropomorphic head phantom (RANDO phantom; Radiology Support Devices, USA) were acquired using a CT scanner (Siemens Healthineers, Germany) at 120 kVp and 209 mAs, with a pixel size of 0.98 × 0.98 mm^2^ and a slice thickness of 2 mm. The acquired images were imported into the kV and MV system TPS, respectively, for target delineation and treatment planning.

#### Target delineation and treatment planning

Eight spherical targets with a diameter of 5 cm were delineated on the CT images of the anthropomorphic head phantom at locations corresponding to the brain, eyeball, nasal cavity, and para-laryngeal regions (Table 1). The target depth was defined as the distance from the phantom surface to the target center along the planned beam direction, ranging from 3.2 to 11.6 cm. Targets #1–#3 in Table 1 were used to evaluate the effects of the target depth on the dosimetric accuracy. The dosimetric effects for targets positioned behind bone (targets #4–#6) and air (target #7) were evaluated. In addition, target #8, located in the nasal cavity, was used to investigate the dosimetric effects of heterogeneous densities.

**Table 1:** Locations of the targets delineated in the anthropomorphic head phantom

Plan	#1	#2	#3	#4	#5	#6	#7	#8
Objective	Depth dependence			Density dependence				
	Deep	Middle	Shallow	High-density bone			Low-density air	Heterogenerous density
Target	#1	#2	#3	#4	#5	#6	#7	#8
Location	Frontal lobe	Frontal lobe	Left eyeball	Cerebrum	Cerebrum	Cerebrum	Para-laryngeal region	Nasal cavity
Depth (cm)	11.6	7.0	3.2	6.4	6.2	4.2	7.0	9.0 (for AP direction)
Field directions	AP	AP	LAO	LT	LAO	LT	LAO	RPO, AP, LPO
Gantry angles	0.0	0.0	45.0	90.0	45.0	90.0	45.0	260.0, 0.0, 100.0

Depth indicates the distance from the phantom surface to the target center measured along the planned beam direction. Plan #8 consisted of three static conformal fields delivered from RPO, AP, and LPO directions.

AP, anterior-posterior; LAO, left-anterior-oblique; LT, left; RPO, right-posterior-oblique; LPO, left-posterior-oblique.

Eight radiotherapy plans were generated for the targets (Table 1). Static conformal radiation fields of 5 × 5 cm^2^ were used in all treatment plans. Plans #1–#7 consisted of a single field, whereas Plan #8 was a 3DCRT plan composed of three fields (Supplementary Fig. 2). The MLC was fitted to the target shape without additional margins, based on the outside edge of the MLC leaves. For each treatment plan, a planned dose of 200 cGy was aimed at the center of mass of the target, which was positioned at the isocenter. The beam directions were selected from various orientations, including anterior-posterior (AP), left-anterior-oblique (LAO), left (LT), right-posterior-oblique (RPO), and left-posterior-oblique (LPO). These treatment plans were generated identically for the kV and MV X-ray beams using the corresponding TPS. Dose calculations were performed using the Equivalent Tissue-Air Ratio and Analytical Anisotropic algorithms for the kV and MV X-ray beams, respectively.

#### Calibration of thermoluminescent dosimeter

TLDs were used for the point-dose measurements because of their small size and tissue-equivalent properties [21]. TLD-100 chips (Thermo Scientific, USA), 3.2 × 3.2 mm^2^ in size and 0.15 mm in thickness, were used.

A TLD calibration procedure was performed to obtain the calibration factors (cGy per signal) for converting TLD signals into absorbed dose and to measure the point doses. Calibration was conducted separately for the kV and MV X-ray beams to account for the energy dependence of the TLD response in the corresponding energy ranges.

The absorbed dose delivered to the TLDs for calibration was quantified by performing dose measurements under the reference conditions using a Farmer-type ionization chamber positioned at the isocenter within a solid water phantom. The reference conditions for the kV X-ray beam were an SSD of 60 cm, a field size of 10 × 10 cm^2^, and a measurement depth of 2 cm; for the MV X-ray beam, the corresponding conditions were an SSD of 100 cm, the same field size, and a measurement depth of 10 cm. A dose of 200 cGy was delivered.

The ionization chamber was then replaced with TLDs, and irradiation was performed under the same conditions. The TLD readout was performed 12 h after irradiation to minimize TLD signal instability. Finally, the TLD signals were background-subtracted and corrected using elemental correction coefficient (ECC, accounting for chip-to-chip sensitivity variations) and a reader calibration factor (RCF, accounting for variations in the reader response between different readout sessions), before conversion to absorbed dose. Based on these procedures, the beam-specific TLD calibration factors were obtained for the kV and MV X-ray beams, respectively.

#### Point dose measurement

The point doses were measured by delivering the planned dose to TLDs positioned at the target locations defined in the treatment plans within the anthropomorphic head phantom. Three TLD chips were packed into a custom three-dimensional (3D)-printed holder, designed to match the same shape and dimensions as the plugs used in the RANDO head phantom (Supplementary Fig. 3). The holder was inserted at the corresponding target location for each plan, and the head phantom was then aligned to the planned treatment position using the X-ray imaging system of the radiotherapy machine. All eight treatment plans were used for TLD dose measurement in the kV and MV X-ray systems, as shown in Table 1. The TLD readout procedure was the same as that described in the above TLD calibration section. The TLD signals were background-subtracted and corrected using ECC/RCF, and the absorbed dose was finally calculated by applying the corresponding TLD calibration factors. For each measurement condition, the measured dose was summarized as the mean ± standard deviation of three TLD chips irradiated under the same condition. The measured point doses were compared with the corresponding doses calculated in the TPS.

#### Statistical analysis

An exact Wilcoxon signed-rank test was performed to compare the absolute percentage difference between the planned and measured doses for the two systems. The median paired difference between systems and its bootstrap 95% confidence interval (CI) were also calculated. These analyses were used because of the small number of paired values (n = 8) and the potential non-normality of the paired differences. All statistical analyses were performed using Python (version 3.14.4) with the pandas (version 3.0.2) and SciPy (version 1.17.1) libraries.

## RESULTS

### Percent depth dose and depth of penetration

Fig. 1 shows the PDD curves measured for the kV and MV X-ray systems. For the 300 kV X-ray beam, the buildup distance was negligible for a small field (4 × 4 cm^2^) and increased to 2 mm for the larger field 15 × 15 cm^2^). In addition, the PDD increased as the field size increased, which was attributed to the increased contribution of scattered X-rays in water.

**Fig. 1:**
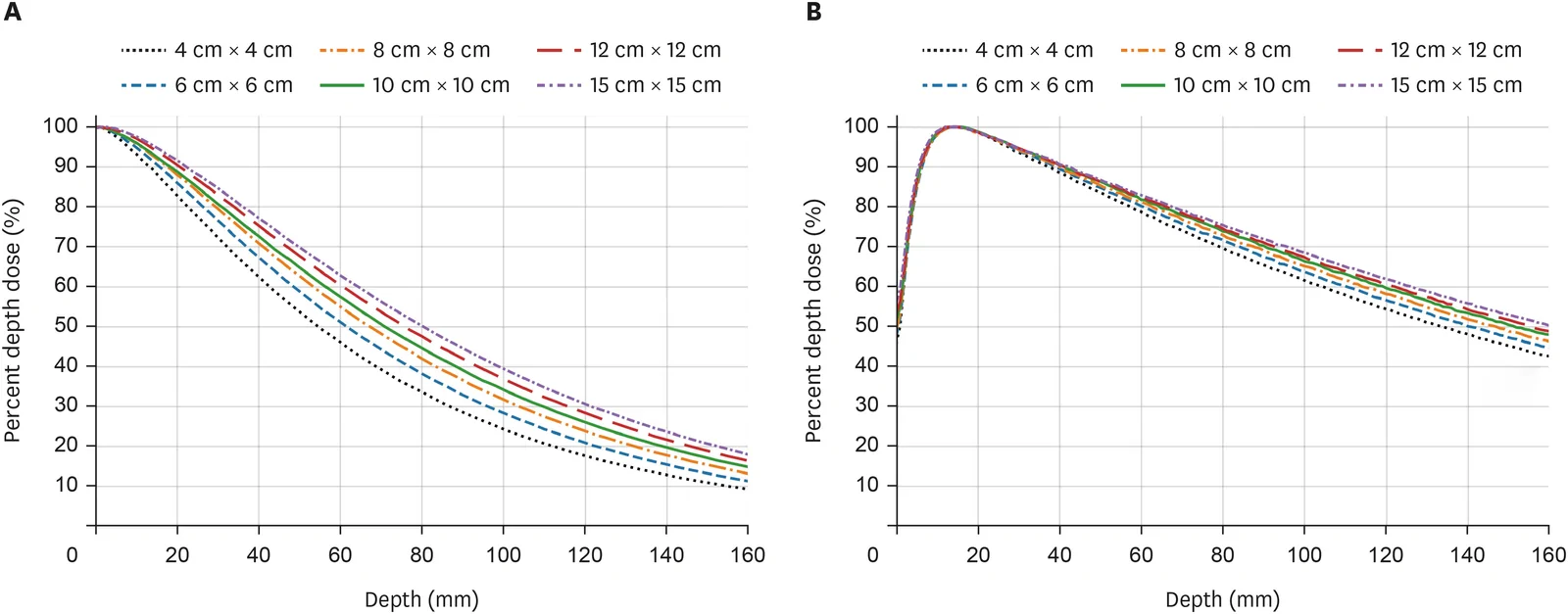
Depth-dose comparison between kilovoltage and megavoltage X-ray beams. Percent depth dose curves measured with different field sizes for (A) the 300 kilovoltage X-ray beam and (B) the 6 megavoltage X-ray beam.

For the 6 MV X-ray beam, the buildup distance increased from 14 mm at 4 × 4 cm^2^ to 16 mm at 15 × 15 cm^2^. Similar to the kV results, an increase in PDD as the field size increased was also observed. The dependence of PDD on the field size was less pronounced than the kV X-ray beam because of the reduced contribution of scattered radiation at higher photon energies.

Fig. 2 shows the depth of dose penetration at various dose levels (50%, 70%, and 90% of the maximum dose) derived from the PDDs for the kV and MV X-ray beams. The penetration depth at the 50% dose level ranged from 54.7 to 80.3 mm for the kV X-ray beam and from 133.7 to 160.7 mm for the MV X-ray beam, depending on the field size. The penetration depth of the MV X-ray beam was approximately 2.45 times greater than that of the kV X-ray beam for the 4 × 4 cm^2^ field size and approximately 2.00 times greater for the 15 × 15 cm^2^ field size, corresponding to the maximum and minimum differences, respectively.

**Fig. 2:**
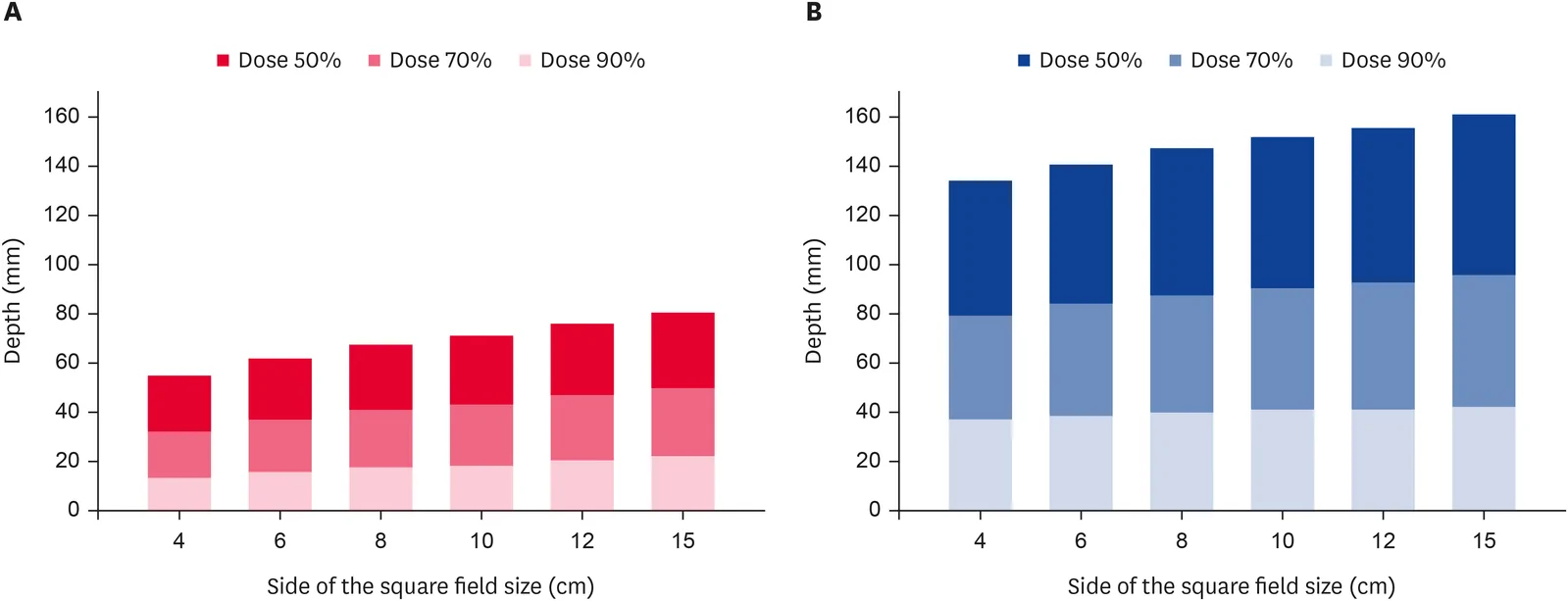
Comparison of dose penetration depths between kilovoltage and megavoltage X-ray beams. Depth of dose penetration at 50%, 70%, and 90% of the maximum dose levels for various field sizes (4 × 4, 6 × 6, 8 × 8, 10 × 10, 12 × 12, and 15 × 15 cm^2^) of (A) 300 kilovoltage X-ray beam and (B) 6 megavoltage X-ray beam. The upper boundary of each colored segment represents the penetration depth corresponding to the 50%, 70%, and 90% dose levels.

### Beam profile

Fig. 3 shows the inline and crossline beam profiles measured for the kV and MV X-ray systems. For the 300 kV X-ray beam, a pronounced heel effect was visually observed in the crossline profile. The flatness and symmetry of the crossline profile were 2.11% and 2.55% higher, respectively, than those of the inline profile, which was unaffected by the heel effect. In addition, the penumbra of the crossline profile was 1.86 mm wider than that of the inline profile.

**Fig. 3:**
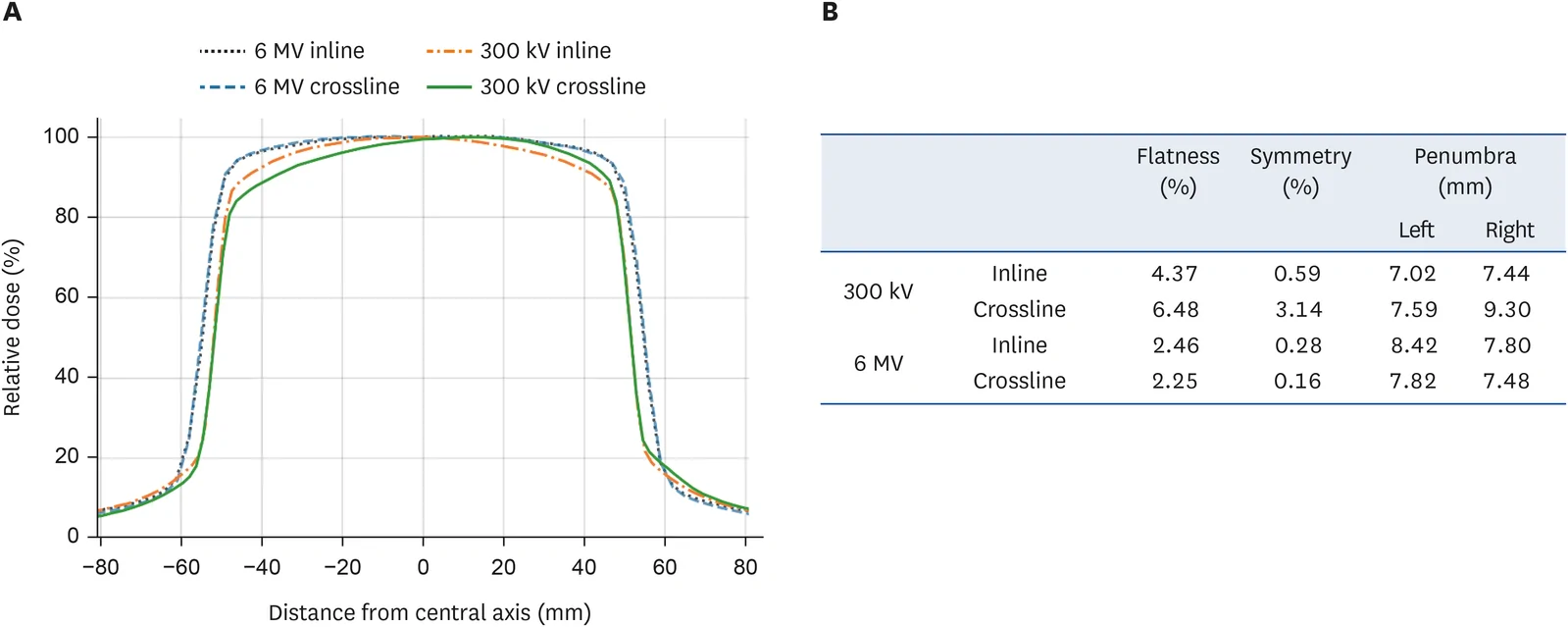
Inline and crossline dose profiles for 300 kV and 6 MV X-ray beams. (A) Profiles measured for a 10 × 10 cm^2^ field at depths of 2 cm for the 300 kV beam and 10 cm for the 6-MV beam. (B) Flatness and symmetry calculated within the central 80% of the full width at half maximum, and left/right penumbra defined as the distance between the 80% and 20% dose levels. kV, kilovoltage; MV, megavoltage.

For the 6 MV X-ray beam, the inline and crossline profiles were visually similar. The differences in flatness and symmetry between the two profiles were within approximately 0.2%, and the maximum difference in penumbra was 0.6 mm.

### Field size output factor

Fig. 4 presents the measured output factors for different field sizes for the kV and MV X-ray beams. For both beams, the output factor increased as the field size was increased. On the other hand, the changes in output factor with field sizes were greater for the kV X-ray beam than for the MV X-ray beam.

**Fig. 4:**
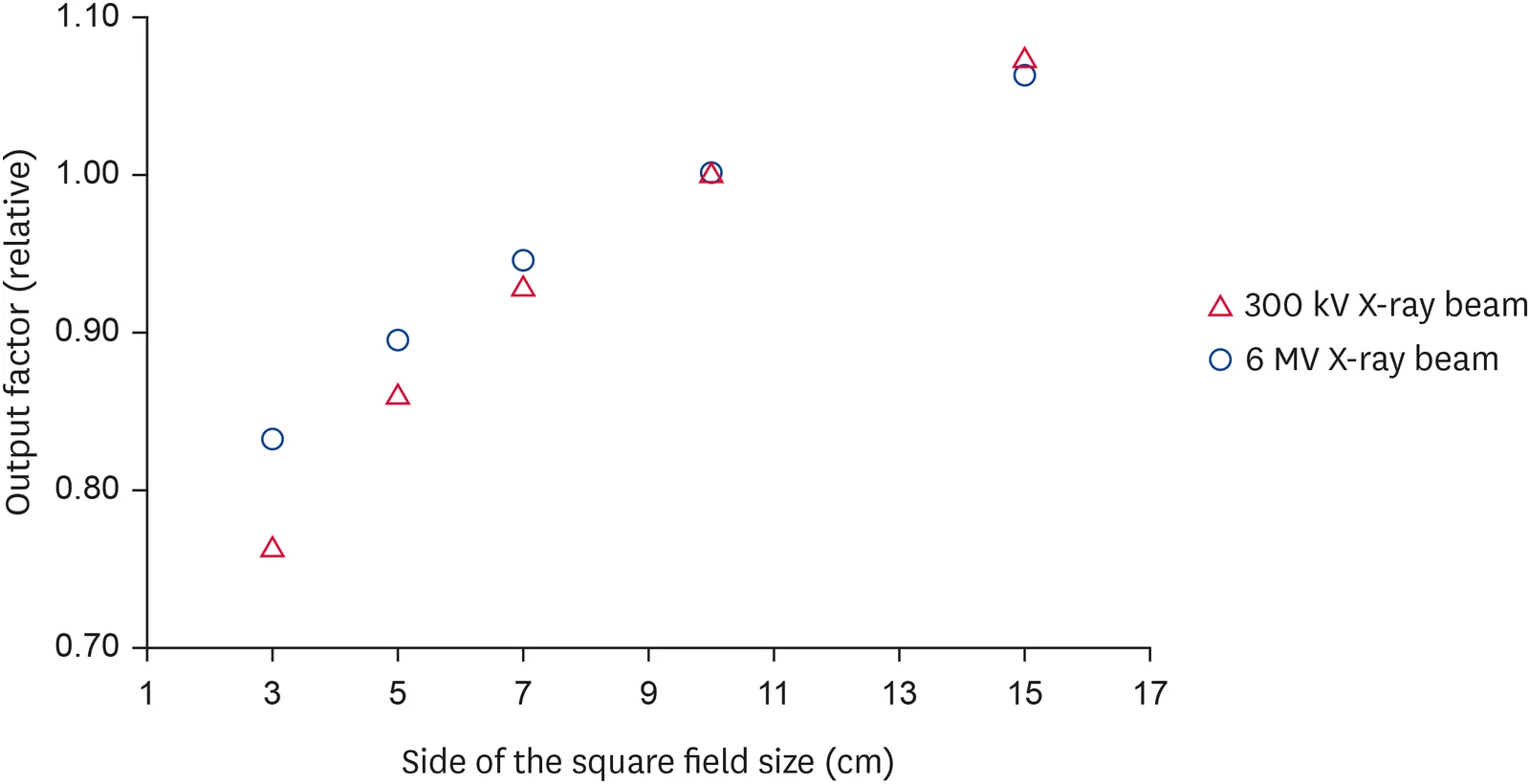
Field size output factors for the kV (red triangles) and MV (blue circles) X-ray beams. Normalization was performed based on the output corresponding to a 10 × 10 cm^2^ field size. kV, kilovoltage; MV, megavoltage.

### Point dose evaluation

Table 2 lists the differences between the calculated and measured point doses for the kV and MV X-ray beams. For the depth-dependence evaluation, the maximum difference was observed at a shallow depth for the kV X-ray beam (4.0%) and at a deep depth for the MV X-ray beam (4.8%), whereas the smallest differences for both systems were observed at middle depths. For the density dependence evaluation, the largest absolute difference was observed behind the high-density bone (2.0% for kV and 6.5% for MV), whereas the corresponding differences behind low-density air and in the heterogeneous-density region were smaller, ranging from 0.7% to 4.1%. For a statistical comparison, the exact Wilcoxon signed-rank test yielded a *p* value of 0.0078. The median paired difference in the absolute percentage difference between the two systems was −1.9 percentage points, with a bootstrap 95% CI of −3.8 to −0.7.

**Table 2:** Comparisons of the planned and measured points doses for kV and MV X-ray beams

Plan		KV X-ray beam			MV X-ray beam		
		Planned dose (cGy)	Measured dose (cGy)	Difference (%)	Planned dose (cGy)	Measured dose (cGy)	Difference (%)
Deep	#1	200.0	207.3 ± 3.8	3.7	200.0	209.6 ± 0.8	4.8
Middle	#2		206.0 ± 3.2	3.0		206.2 ± 2.3	3.1
Shallow	#3		207.9 ± 8.7	4.0		209.4 ± 18.9	4.7
High-density bone	#4	200.0	200.2 ± 1.8	0.1	200.0	209.7 ± 2.6	4.9
	#5		196.0 ± 1.7	–2.0		213.0 ± 3.1	6.5
	#6		202.5 ± 4.8	1.3		206.6 ± 6.2	3.3
Low-density air	#7	200.0	201.3 ± 6.1	0.7	200.0	206.7 ± 0.4	3.4
Hetero generous density	#8	200.0	204.6 ± 5.5	2.3	200.0	208.1 ± 2.4	4.1

Measured doses are presented as mean ± standard deviation of three TLD chips irradiated simultaneously for each plan (n = 3).

kV, kilovoltage; MV, megavoltage; TLD, thermoluminescent dosimeter.

## DISCUSSION

This paper is the first to present a measurement-based dosimetric comparison between a companion animal–dedicated kV X-ray radiotherapy system and an MV LINAC system. This study evaluated the basic beam characteristics, including PDDs, depth of dose penetration, beam profiles, and field size output factors, for both systems. The dose delivery accuracy was also assessed through point dose measurements. Unlike the MV X-ray beam, the kV X-ray beam showed a negligible buildup distance. The penetration depth of the kV X-ray beam was substantially lower than that of the MV X-ray beam, with a maximum difference of up to 2.45 times at the 50% dose level. The field size dependence of the PDD and output factors was more pronounced for the kV X-ray beam than for the MV X-ray beam. Owing to the heel effect, the kV X-ray beam exhibited poorer flatness and symmetry, along with a wider penumbra, in the crossline beam profile than the MV X-ray beam, whereas the inline beam profiles were comparable between the two systems. Despite these differences in beam characteristics, the point-dose measurements showed absolute differences of 4.0% or less between the calculated and measured doses for the kV X-ray beam. These findings suggest that the kV X-ray beam model was appropriately commissioned for the dose calculation across the evaluated target-depth and tissue-density conditions.

The maximum difference between the calculated and measured point doses was observed for the MV beam behind bone, which may have been influenced by measurement-related factors, including uncertainties in TLD readout, phantom setup, and TLD positioning. A previous study using an anthropomorphic phantom showed that TLD-based verification can be affected by such uncertainties [22]. In that study, the overall variation of TLD measurements ranged from 0.9% to 5.9%, depending on the TLD location within the anthropomorphic phantom. In the present study, the TLDs were calibrated separately for the kV and MV beams. Under these conditions, the effect of beam energy on TLD sensitivity was likely minor. Scarboro et al. [23] reported that the TLD response was not substantially affected by variations in photon energy spectra for most in-field measurements with MV beams. The statistical analysis showed a significant difference in the absolute percentage differences between the two systems. Nevertheless, this finding does not necessarily indicate intrinsically higher dosimetric accuracy for the kV system than for the MV system because measurement-related uncertainties may also have contributed to this result.

The main differences in basic beam characteristics between the kV and MV X-ray beams were 1) buildup/dose penetration, 2) field-size dependence, and 3) the heel effect. The first difference is inherently related to the photon energy [10]. The second difference can be attributed to the increased contribution of scattered radiation as the field size increases. Lower-energy photons are more likely to undergo scattering interactions in water, and this scattered dose contributes to the measured dose [24]. The heel effect arises from the geometry of kV X-ray beam generation and is difficult to eliminate, even with filtration.

Among these differences, the heel effect is particularly important in treatment planning. The observed dose calculation accuracy suggests that the TPS beam model for the kV system appropriately accounted for this beam characteristic. On the other hand, this does not indicate that the nonuniformity in the crossline beam profile caused by the heel effect has been eliminated. Therefore, for relatively large targets or multi-field delivery, hot spots may occur depending on the beam orientation, beam overlap pattern, and beam weighting. In 3DCRT using forward planning, such hot spots should be managed carefully during treatment planning and plan evaluation. If IMRT or VMAT becomes available for the kV system in the future, such hot spots may be more easily minimized through inverse optimization.

The buildup distances obtained from the PDDs of the kV X-ray beam measured in this study were comparable to those calculated using MC simulations reported by Seo et al. [12], but the measured penetration depth was greater. This difference can be attributed to 1) differences in the generated X-ray energy spectrum and 2) differences in the filtration used for beam hardening. Consequently, the kV X-ray system investigated in this study showed deeper penetration, suggesting that improved treatment planning may be achievable compared to previous simulation-based studies.

Despite the favorable performance of the kV X-ray system observed in this study, the system has several limitations: 1) a negligible buildup distance, 2) limited penetration depth, and 3) higher bone dose. The first of these limitations results in a lack of skin-sparing effect [25], potentially leading to skin toxicity such as burns, during radiotherapy. Veterinary-specific quantitative skin dose limits are not yet well established. Therefore, skin protection should be based on minimizing the field size and beam overlap on the same skin region, together with close monitoring of acute skin reactions. *In vivo* dosimetry may also be useful for patient-specific skin dose monitoring, particularly to detect unexpectedly high surface doses [26,27]. In such cases, treatment plan modification, including rearrangement of beam directions, may be considered to reduce the surface dose. One potential technique for reducing the surface dose is minibeam irradiation, in which radiation is delivered through arrays of spatially fractionated, narrow beamlets. Recent studies have revealed the skin-sparing effects of this technique for photon and proton beams [28,29]. The application of minibeam techniques to the kV X-ray system may help address this limitation in the future.

The second limitation may hinder the treatment of deeply located tumors, despite companion animals being smaller than humans, because of their compact, complex anatomy, in which bony structures, soft tissues, and organs are arranged within a relatively small body. Under such conditions, treatment planning with kV beams may require careful consideration of scatter and tissue heterogeneity. This may limit the applicability of kV beams to deeply located tumors compared with MV beams. Nevertheless, several recent MC simulation studies have suggested that deeply located tumors may be treated using kV X-ray beams with arc therapy techniques [11,30,31]. Although these findings suggest a potential role for kV beams in selected deeply located tumors, such delivery techniques are unavailable in the present kV X-ray system. Therefore, kV beams may be considered mainly for targets located at shallow depths.

The third limitation is the increased absorbed dose to bone at kV energies due to the photoelectric effect. This may increase the risk of bone-related toxicity such as osteonecrosis. Although agreement between calculated and measured point doses was observed for targets located behind these bony structures, this finding does not exclude the possibility of bone-related toxicity. In addition, although beam hardening may shift the effective photon spectrum toward higher energies, this does not necessarily mean that bone-related dose effects are reduced. Therefore, further assessment of the bone dose and its potential clinical impact is needed.

A primary limitation of the present study is the use of a human RANDO phantom for point-dose verification. The attenuation and scatter/backscatter conditions around the measurement point of the phantom may differ from those in a veterinary phantom because the phantom does not fully reproduce the skeletal structure, tissue thickness, and local arrangement of bone, air cavities, and soft tissue in companion animals. This may introduce uncertainty in the absolute dose measurements, particularly in kV X-ray dosimetry. For example, full backscatter conditions may be achieved in a relatively larger and thicker human phantom, whereas this may not be the case in a smaller veterinary phantom. This difference could lead to a lower measured dose in the veterinary phantom. A previous study showed that insufficient backscatter can affect the measured dose depending on the phantom thickness and field size [32]. Thus, a further study using a veterinary phantom will be needed to account for these effects.

Another limitation is that dose accuracy was evaluated using point-dose measurements. Accuracy evaluations based on 2D or 3D dose distributions will be needed to address this limitation. On the other hand, 2D dosimeters such as radiochromic films are challenging to position at specific locations, such as bone-equivalent regions, within an anthropomorphic head phantom. In addition, 3D dosimeters, such as gel dosimeters, remain limited for routine use because of their sensitivity to environmental factors such as temperature. Therefore, novel 2D and 3D dosimeters are required to achieve accurate dose evaluations, which is beyond the scope of the present study.

The kV and MV X-ray systems differed in their dosimetric characteristics. Despite these differences, the kV X-ray system showed agreement between the calculated and measured point doses under various depth and density conditions. These findings suggest that kV X-ray systems can achieve accurate dose calculations when the treatment planning system is properly commissioned using measured beam data. Owing to the limited penetration depth of kV beams, their application may be considered mainly for targets located at shallow depths in companion animal radiotherapy.

## SUPPLEMENTARY MATERIALS

Supplementary Fig. 1 Photograph of the kilovoltage and megavoltage X-ray radiotherapy systems. (A) LEP 300 system and (B) TrueBeam linear accelerator.

Supplementary Fig. 2 Comparison of planned dose distributions. Examples of 3D conformal radiotherapy plans in (A) the kilovoltage X-ray system and (B) the megavoltage X-ray system. (C) 3D views of the treatment plans and corresponding phantom setup.

Supplementary Fig. 3 TLD holder assembly and insertion into the head phantom. (A) TLDs inserted into a 3D-printed holder, (B) the fully assembled holder, and (C) the assembled holder inserted into the head phantom.
